# Supplementary material for: MetaRibo-Seq measures translation in microbiomes
Source: Nat Commun. 2020 Jun 29;11:3268. doi: 10.1038/s41467-020-17081-z (PMC7324362; doi:10.1038/s41467-020-17081-z)
Supplement: Supplementary file 10 — Supplementary Data 7 [file 41467_2020_17081_MOESM10_ESM.zip › File2/Confidence_High_Taxonomy/378625_out.krona.html]

Javascript must be enabled to view this page.

members
magnitude
magnitudeUnassigned
count
unassigned
taxon
rank

378625\_out

3

superkingdom
3
2

1239
3
phylum

class
3
186801

3
186802
order

family
3
541000

genus
1263
3

species

SRS014235\_contig\_number\_35831SRS024492\_contig\_number\_12394SRS052697\_contig\_number\_33758
46228
3
